# Effect of Ezetimibe on LDL-C Lowering and Atherogenic Lipoprotein Profiles in Type 2 Diabetic Patients Poorly Controlled by Statins

**DOI:** 10.1371/journal.pone.0138332

**Published:** 2015-09-23

**Authors:** Kentaro Sakamoto, Mitsunobu Kawamura, Takahide Kohro, Masao Omura, Takayuki Watanabe, Keiko Ashidate, Toshiyuki Horiuchi, Hidehiko Hara, Nobuo Sekine, Rina Chin, Motoyoshi Tsujino, Toru Hiyoshi, Motoki Tagami, Akira Tanaka, Yasumichi Mori, Takeshi Inazawa, Tsutomu Hirano, Tsutomu Yamazaki, Teruo Shiba, RESEARCH Study Group

**Affiliations:** 1 Toho University Ohashi Medical Center, Department of Diabetes and Metabolism, Tokyo, Japan; 2 Tokyo Teishin Hospital, Division of Endocrinology and Metabolism Department of Internal Medicine, Tokyo, Japan; 3 Jichi Medical University, Department of Medical Informatics / Cardiology, Tochigi, Japan; 4 Yokohama Rosai Hospital, Department of Endocrinology and Metabolism, Kanagawa, Japan; 5 Yokohama City Minato Red Cross Hospital, Department of Internal Medicine, Kanagawa, Japan; 6 Kudanzaka Hospital, Department of Internal Medicine, Tokyo, Japan; 7 Tokyo Metropolitan Health Medical Treatment Corporation Toshima Hospital, Department of Endocrinology and Metabolism, Tokyo, Japan; 8 Toho University Ohashi Medical Center, Department of Cardiology, Tokyo, Japan; 9 Tokyo Koseinenkin Hospital, Department of Internal Medicine, Tokyo, Japan; 10 Tokyo Kyosai Hospital, Department of Internal Medicine, Tokyo, Japan; 11 Tokyo Metropolitan Tama Medical Center, Department of Internal Medicine, Tokyo, Japan; 12 Japanese Red Cross Medical Center, Tokyo, Japan; 13 Sanraku Hospital, Life-style related Disease Clinic, Tokyo, Japan; 14 Kagawa Nutrition University, Nutrition Clinic, Tokyo, Japan; 15 Toranomon Hospital, Department of Endocrinology and Metabolism, Tokyo, Japan; 16 Kashiwa City Hospital, Internal Medicine, Chiba, Japan; 17 Showa University School of Medicine, Department of Medicine Division of Diabetes Metabolism and Endocrinology, Tokyo, Japan; 18 The University of Tokyo Hospital, Clinical Research Support Center, Tokyo, Japan; 19 Mitsui Memorial Hospital, Division of Diabetes and Metabolism, Tokyo Japan; Campus Bio-Medico University, ITALY

## Abstract

**Background:**

There exists a subpopulation of T2DM in whom first-line doses of statin are insufficient for optimally reducing LDL-C, representing a major risk of CVD. The RESEARCH study focuses on LDL-C reduction in this population along with modifications of the lipid profiles leading to residual risks.

**Methods:**

Lipid changes were assessed in a randomized, multicenter, 12-week, open-label study comparing a high-potency statin (10mg of atorvastatin or 1mg of pitavastatin) plus ezetimibe (EAT: n = 53) with a double dose of statin (20mg of atorvastatin or 2mg of pitavastatin) (DST: n = 56) in DM subjects who had failed to achieve the optimal LDL-C targets. Lipid variables were compared with a primary focus on LDL-C and with secondary focuses on the percentage of patients who reached the LDL-C targets and changes in the levels of RLP-C (remnant like particle cholesterol) and sd-LDL-C, two characteristic atherogenic risks of DM.

**Results:**

The reduction of LDL-C (%), the primary endpoint, differed significantly between the two groups (-24.6 in EAT vs. -10.9 in DST). In the analyses of the secondary endpoints, EAT treatment brought about significantly larger reductions in sd-LDL-C (-20.5 vs. -3.7) and RLP-C (-19.7 vs. +5.5). In total, 89.4% of the patients receiving EAT reached the optimized treatment goal compared to 51.0% of the patients receiving DST. The changes in TC (-16.3 vs. -6.3) and non-HDL-C (-20.7 vs. -8.3) differed significantly between the two groups.

**Conclusion:**

Ezetimibe added to high-potency statin (10 mg of atorvastatin or 1 mg of pitavastatin) was more effective than the intensified-dose statin (20 mg of atorvastatin or 2 mg of pitavastatin) treatment not only in helping T2DM patients attain more LDL-C reduction, but also in improving their atherogenic lipid profiles, including their levels of sd-LDL-C and RLP-C.

We thus recommend the addition of ezetimibe to high-potency statin as a first line strategy for T2DM patients with insufficient statin response.

**Trial Registration:**

The UMIN Clinical Trials Registry UMIN000002593

## Introduction

Patients with dyslipidemia complicated by diabetes are highly prone to cardiovascular disease and mortality [1, 2]. Many guidelines for atherosclerotic diseases [3, 4] have supported the inclusion of patients with diabetes in the high-risk category, confirmed the benefits of LDL-lowering therapy, and lowered the target value for prevention in these patients. Yet only around half of diabetic patients meet their LDL-C targets in the U.S. [5] and Japan [6, 7, 8]. Recent surveys have shown that the patients at the highest cardiovascular risk more often fail to achieve their therapeutic goal, especially diabetics [9]. The use of super-high-dose statin treatments to achieve LDL-C goals leads to more frequent side effects and more persistent cardiovascular risk. These circumstances evoke the demand for investigating the possibility and establishing alternative LDL-cholesterol-lowering treatments other than increasing in first-choice statin doses.

Moreover, there remain certain risks not fully accounted for by the decreases in LDL cholesterol. Potential culprits include insulin resistance, metabolic syndrome, and an abnormal lipid profile (e.g., inappropriate levels of small dense LDL (sd-LDL) and remnant lipoproteins), a significant risk factor for coronary heart disease [10–13]. Small dense LDL cholesterol (sd-LDL-C) manifesting as a change in LDL particle size has also been recognized as an emerging cardiovascular risk factor, one for which screening is recommended in patients at high risk for cardiometabolic disorders [13].

Recent years have seen the emergence of a novel strategy for lowering cholesterol by inhibiting cholesterol absorption with ezetimibe blocking Niemann-Pick C1-like 1 [14]. As statins increase the absorption of fractional cholesterol, a combination therapy with cholesterol absorption inhibitors is therefore thought to be a promising and feasible strategy especially for diabetics, a population in whom upregulation of NPC1L1 has been observed [15].

Our group designed RESEARCH (Recognized Effect of Statin and Ezetimibe therapy for achieving LDL-C Goal) [16] as a randomized, doctor-oriented, multicenter trial to compare the effects of higher-dose statin versus ezetimibe-plus-statin on the serum LDL-C concentration of type 2 diabetes patients with a wide range of clinical backgrounds. This study investigates type 2 diabetic patients with hyper LDL-cholesterolemia who cannot achieve their LDL-C targets with the first-line doses of high-potency statin. We will evaluate the effect of ezetimibe-add-on therapy in comparison to the conventional strategy of intensified high-potency statin by investigating changes in lipid profiles and defining the rate of LDL-C change as the primary endpoint.

## Methods

**Fig 1:**
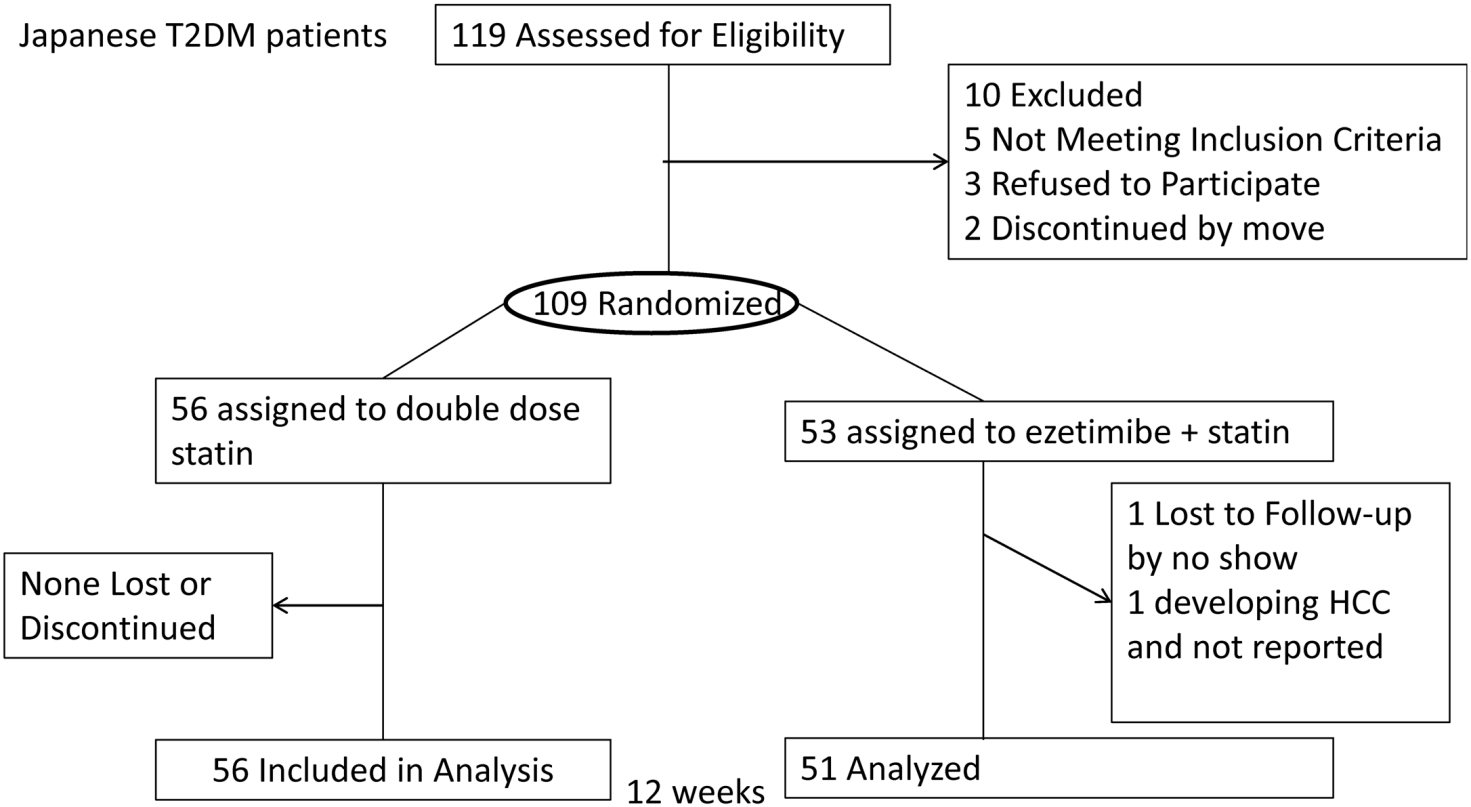
Flow of Patients in the RESEARCH Study. One hundred and nine patients were randomized. Fifty-six patients were assigned to the double intensified dose statin therapy (DST) group and 53 patients were assigned to the ezetimibe-add-on therapy (EAT) group. The analysis was performed on 51 patients in the EAT group and 56 patients in the DST group. T2DM: type 2 diabetes mellitus.

The RESEARCH study is a prospective, randomized, multicenter, clinical trial conducted to examine whether a combination of high-potency statins (10 mg of atorvastatin or 1 mg of pitavastatin) plus ezetimibe lowers the serum LDL-C concentration in type 2 diabetes outpatients compared with intensified high-potency statins such as atorvastatin and pitavastatin (20 mg of atorvastatin or 2 mg of pitavastatin). The RESEARCH protocol has been presented as a CONSORT diagram (Fig 1) and described in detail elsewhere [16]. The protocol is shown again as supplementary information online (S1 and S2 Protocols, S1 CONSORT Checklist). Briefly, this study is being undertaken in accordance with the Declaration of Helsinki and guidelines from the Japanese Ministry of Health, Labour and Welfare (complete revision on December 28, 2004). Every participating center in Japan has obtained approval for the study by a local research ethics committee (the ethics committee of Toho University Ohashi Medical Center; the Research Ethics Committee of Mitsui Memorial Hospital; the Institutional Review Board of Yokohama City Minato Red Cross Hospital; the ethics committee of Kudanzaka Hospital; the Institutional Review Board of Tokyo Metropolitan Tama Medical Center; the ethics committee(s) of Tokyo Teishin Hospital; the ethics committee of Japan Community Healthcare Organization, Tokyo Shinjuku Medical Center; the ethics committee of Sanraku Hospital; the Institutional Review Board of Kashiwa City Hospital; and the Institutional Review Board of Toranomon Hospital and Toranomon Hospital Kajigaya). All of the patients have given their fully informed written consent.

### Patients

At the time of enrollment, the type 2 diabetic outpatients were over 20 years of age and had failed to reach the target LDL-C values recommended by the guideline [17] (LDL-C < 120 mg/dL for patients with no history of CAD; LDL-C < 100 mg/dL for patients with a history of CAD) after receiving high-potency statins (10 mg of atorvastatin or 1 mg of pitavastatin) for more than 1 month. In Asia, these doses of high-potency statins would bring stronger response and attain more than 30% reduction of LDL-C in contrast to Caucasians. [18–20]

### Randomization and study treatment

The patients were allocated to two groups by dynamic allocation methods, one for the ezetimibe-add-on therapy (the EAT group) and the other for the intensified statin therapy to double dose (the DST group). Randomization was performed with stratification according to age and gender. When a patient was enrolled, a doctor placed an order for random assignment by entering the data (including age and year) into the randomization software installed at the monitoring office of Nouvelle Plus. The patients assigned to the DST group received high-potency statins at double the baseline dose (atorvastatin doubled from 10 mg to 20 mg, pitavastatin doubled from 1 mg to 2 mg). The patients assigned to the EAT group received 10 mg/day of ezetimibe in addition to their high-potency statin at the baseline dose (10 mg of atorvastatin or 1 mg of pitavastatin).

### Outcome measures

The primary end point was the percent change in the level of LDL-C from baseline to week 12 of treatment. Sd-LDL and remnant-like particle cholesterol (RLP-C) were measured to evaluate the residual risks after the intervention into LDL-cholesterol with high-potency statins, and their percent changes were defined as major secondary end points. Other pre-specified secondary endpoints included the rates at which the target LDL-C values recommended by the guidelines [17] were achieved and the percent changes in total cholesterol (TC), triglyceride (TG), high-density lipoprotein cholesterol (HDL-C), high-sensitivity C-reactive protein (HS-CRP), and HbA1c. LDL-C values were determined by Friedelwald’s formula (LDL-C = TC—HDL-C—TG/5). Each institution measured TC, HDL-C, and TG and performed general laboratory tests to determine parameters such as HbA1c, glucose, AST, ALT, creatinine, and CPK. Non-HDL-cholesterol was calculated by the following formula: Non-HDL-C = TC—HDL-C. Sd-LDL was measured at Showa University (Tokyo, Japan) by the precipitation method previously described [21]. RLP-C was measured by SRL, Japan.

### Statistical analysis

The initial estimate for the required sample size was 60 subjects per group. This estimate was derived from the following hypothesis: a doubled statin dose leads to a 11.7% reduction in LDL-C beyond the initial dose of statin, whereas the addition of ezetimibe leads to a 21.7% reduction in LDL-C (standard deviation: 15%). The significance level was set at 5%, the power was set at 90%, and the follow-up ratio was estimated to be 80%.

In the assessments for the end points, differences between the two groups in categorical variables were evaluated using the chi-square tests and differences in continuous variables were evaluated using the Wilcoxon tests. The Wilcoxon signed-rank tests were used to assess the differences in parameters before and after treatment within each group, except for age. A p value of less than 0.05 (2-sided) was assumed to indicate significance in all analyses. All data were analyzed using Stata version 12.1 (StataCorp LP, Texas, U.S.A.).

## Results

One hundred and nine diabetic subjects with a wide range of clinical backgrounds were recruited (63 men and 46 women). Of these, 53 patients were assigned to the EAT group and 56 patients were assigned to the DST group. Data at 12 weeks were obtained from 56 patients in DST group, and 51 patients in EAT group (S1 Table). No significant differences between the groups were found in the demographic profiles, coronary risks, or laboratory data, though slight differences were found in the levels of non-HDL-C, apo-B, and sd-LDL (Tables 1 and 2).

**Table 1:** Demographic profiles of treatment groups.

		DST (n = 56)	EAT (n = 53)	p (t or chi-square)
**Age**	**years**	**62.6 ±9.5 (56)**	**61.7 ±11.1 (53)**	**0.673**
**male gender**	**male**	**57.1(32)**	**58.5 (31)**	**0.887**
**Smoking**	**%**	**23.6 (13)**	**24.5 (13)**	**0.617**
**History of Stroke**	**%**	**3.6% (2)**	**0.0 (0)**	**0.169**
**History of CHD**	**%**	**10.7 (6)**	**15.1 (8)**	**0.495**

**Table 2:** Baseline Characteristics of treatment groups.

		DST (n = 56)	EAT (n = 53)	p (Wilcoxon)
**TC**	**mg/dl**	**219±27**	**211±29**	**0.206**
**TG**	**mg/dl**	**162±88**	**147±95**	**0.18**
**HDL-C**	**mg/dl**	**54.7±9.6**	**56.7±15.2**	**0.973**
**LDL-C**	**mg/dl**	**132±24**	**126±21**	**0.288**
**non HDL-C**	**mg/dl**	**164±25**	**155±27**	**0.045**
**RLP-C**	**mg/dl**	**6.35±3.95**	**6.11± 4.80**	**0.368**
**Apo B**	**mg/dl**	**111± 18**	**102± 16**	**0.015**
**MDA-LDL**	**mg/dl**	**157±62**	**113±28**	**0.055**
**sd-LDL-C**	**mg/dl**	**52.2±17.9**	**46.2±16.0**	**0.048**
**hs-CRP**	**mg/L**	**1.11 ± 149**	**0.92±112**	**0.603**
**HbA1c**	**%**	**7.26±0.97**	**7.24±0.65**	**0.692**
**AST**	**U/L**	**23.1±12.2**	**23.7±12.2**	**0.854**
**ALT**	**U/L**	**23.6±10.9**	**24.7±12.5**	**0.832**
**CPK**	**U/L**	**117±58**	**123±88**	**0.964**

### Primary end point

**Fig 2:**
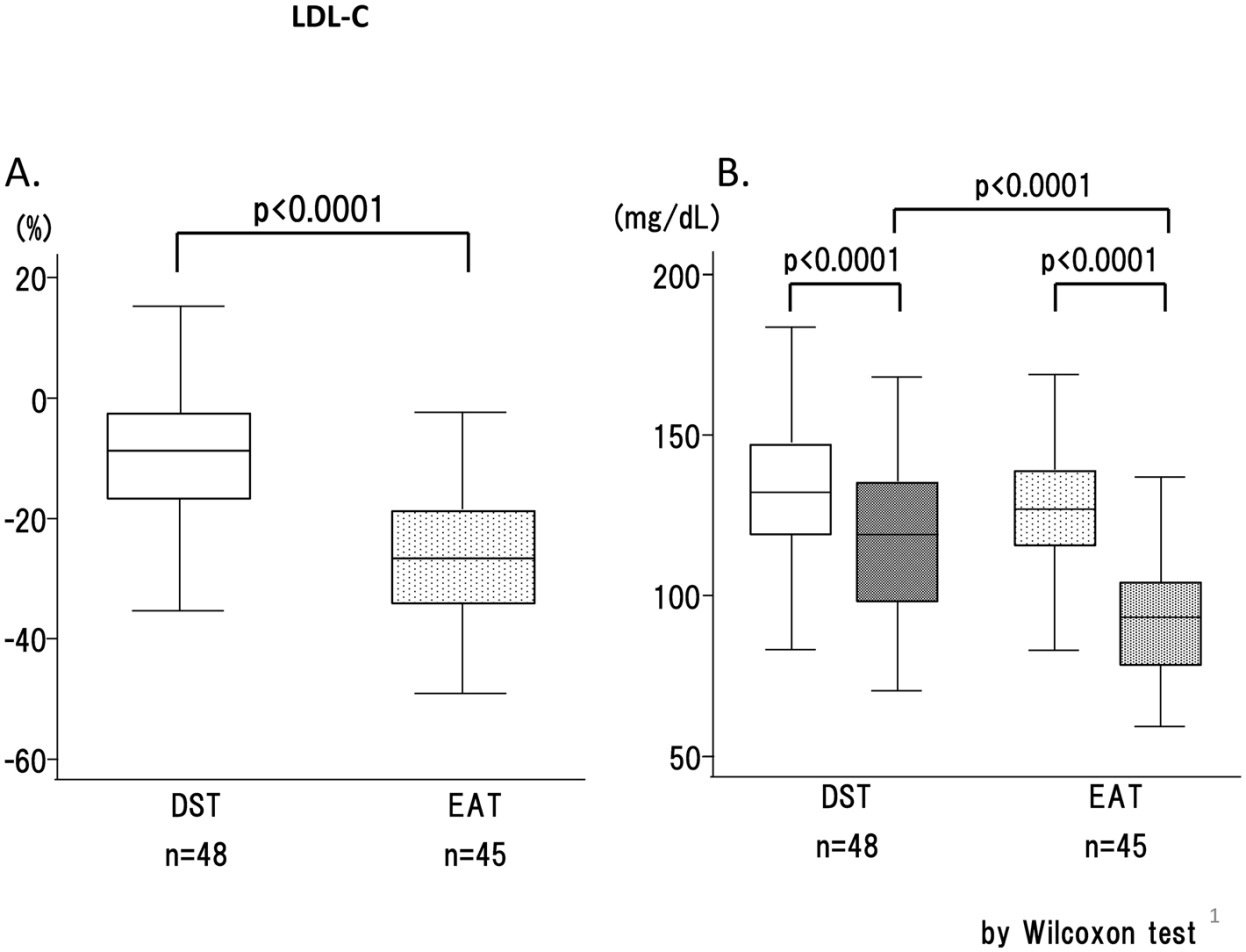
Changes in LDL cholesterol (box plots). **A. Percentage reduction of LDL-C after 12 weeks of treatment:** The solid bar represents the percent change in DST and the dotted bar represents the percent change in EST. B. **LDL-C values before and after 12 weeks of treatment:** The white bars represent basal values before treatment and the black bars represent values after treatment. The dotted textile indicates EAT therapy and the solid bar indicates DST therapy. n.s., not significant; *, p < 0.05; **, p < 0.01; ***, p < 0.001; ****, p < 0.0001.

The primary end point, the percent change in LDL-C levels from baseline to week 12, was significantly greater in the EAT group (-24.6%) than in the DST group (-10.9%), as shown in Fig 2A (p = 0.0000036). The reduction attained in the EAT group was more than double that attained in the DST group.

### Secondary end point

**Fig 3:**
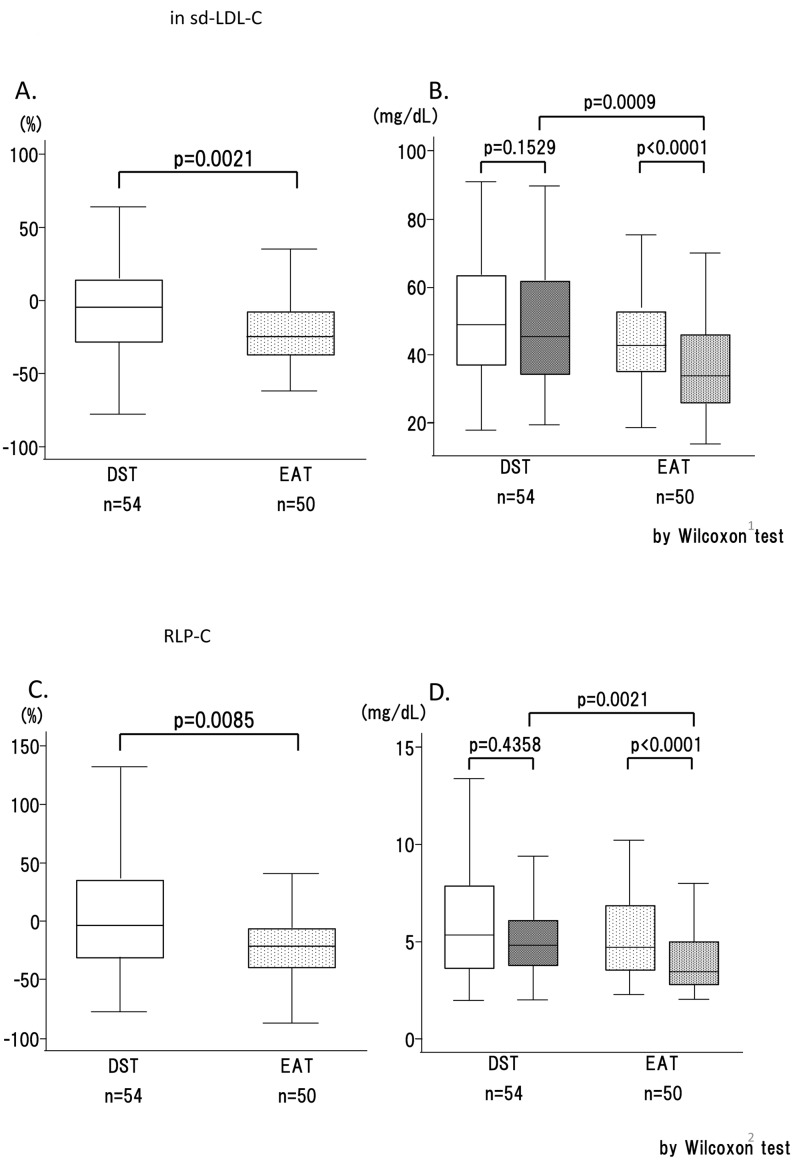
The changes in atherogenic lipid profiles (box plots). A, C: The percent changes in sd-LDL-C and RLP-C, respectively. B, D: sd-LDL-C and RLP-C values before and after the 12 weeks of treatment, respectively. The ‘before treatment’ values are shown as white bars and the ‘after treatment’ values are shown as black bars. n.s., not significant; *, p < 0.05; **, p < 0.01; ***, p < 0.001; ****, p < 0.0001.

**Fig 4:**
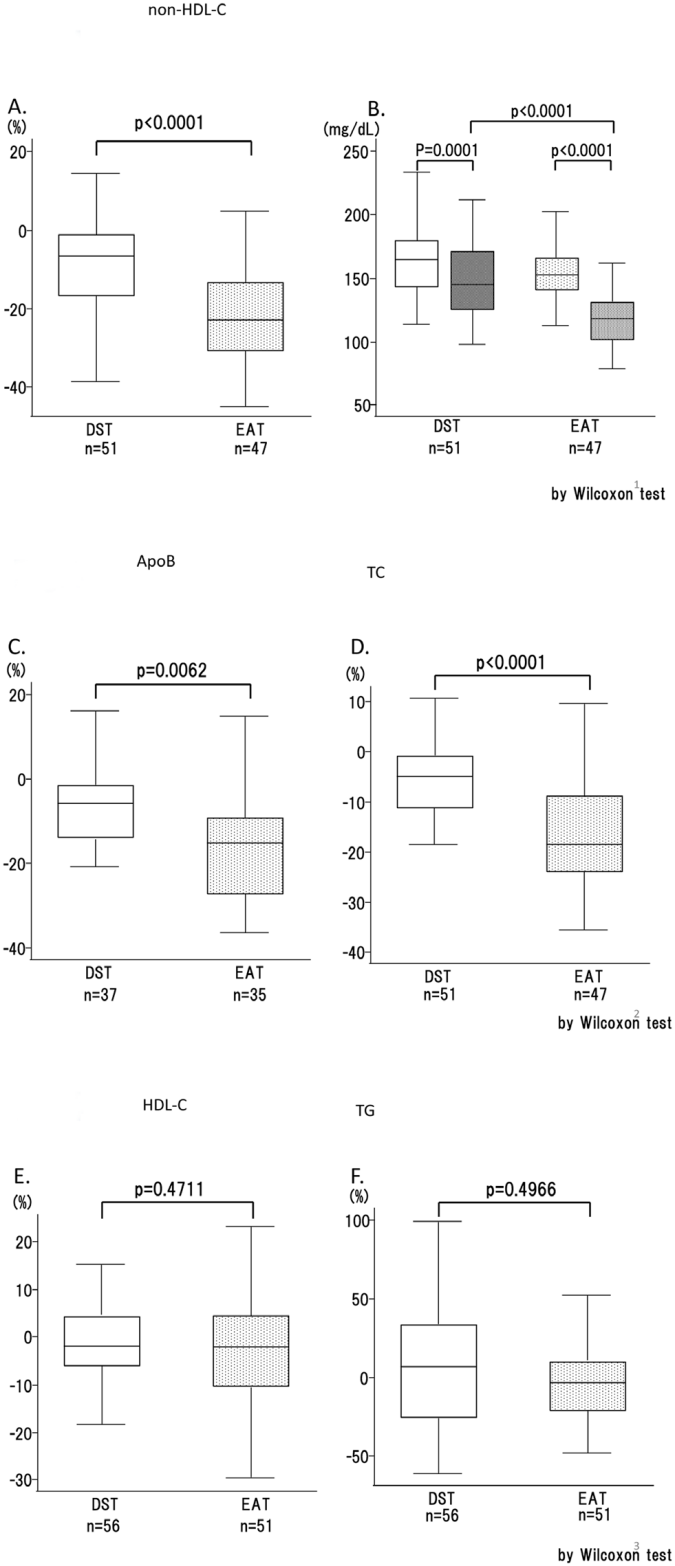
Changes in basic lipid biochemistry (box plots). A, C: The percent changes in non-HDL-C and ApoB, respectively. B: Non-HDL-C values before and after the 12 weeks of treatment. D, E, F: The percent changes in TC, HDL-C, and TG, respectively. The ‘before treatment’ values are shown as solid bars and the ‘after treatment’ values are shown as dotted bars. The bars representing the DST group are white and the bars representing the EAT group are black. n.s., not significant; *, p < 0.05; **, p < 0.01; ***, p < 0.001; ****, p < 0.0001.

Smaller and denser LDL particles are more strongly indicative of CVD risk than large buoyant LDL. Here we used a homogeneous assay for sd-LDL-C [21]. A prominent and significant difference was observed in the percent change of sd-LDL-C: -20.5% in EAT vs. -3.7% in DST (p = 0.0021) (Fig 3A). The mean sd-LDL-C value in DST fell from 52.1±18.2 mg/dl to 48.3±19.4 mg/dl, while that in EAT fell from 45.8±15.9 mg/dl to 35.7 ±14.6 mg/dl (Fig 3B), a value approaching the cut-off point for sd-LDL-C in both Japan (35 mg/dl or 0.9 mmol/l) and the U.S. (40 mg/dl or 1.0 mmol/l) [22]. The EAT subjects exhibited a significantly larger change than the DST subjects in the analyses of RLP-C (19.7% decrease in EAT vs. a 5.5% increase in DST) (p = 0.0085) (Fig 3C), and the difference was confirmed by the actual changes measured (Fig 3D). The EAT treatment brought about a greater reduction of non-HDL-C than the DST treatment (-20.7% vs. -8.3%), with a larger value change (Fig 4A and 4B). Regarding hs-CRP, another predictor for CVD and MDA-LDL, no significant changes were observed in either group and no difference was observed between groups (data not shown).

**Fig 5:**
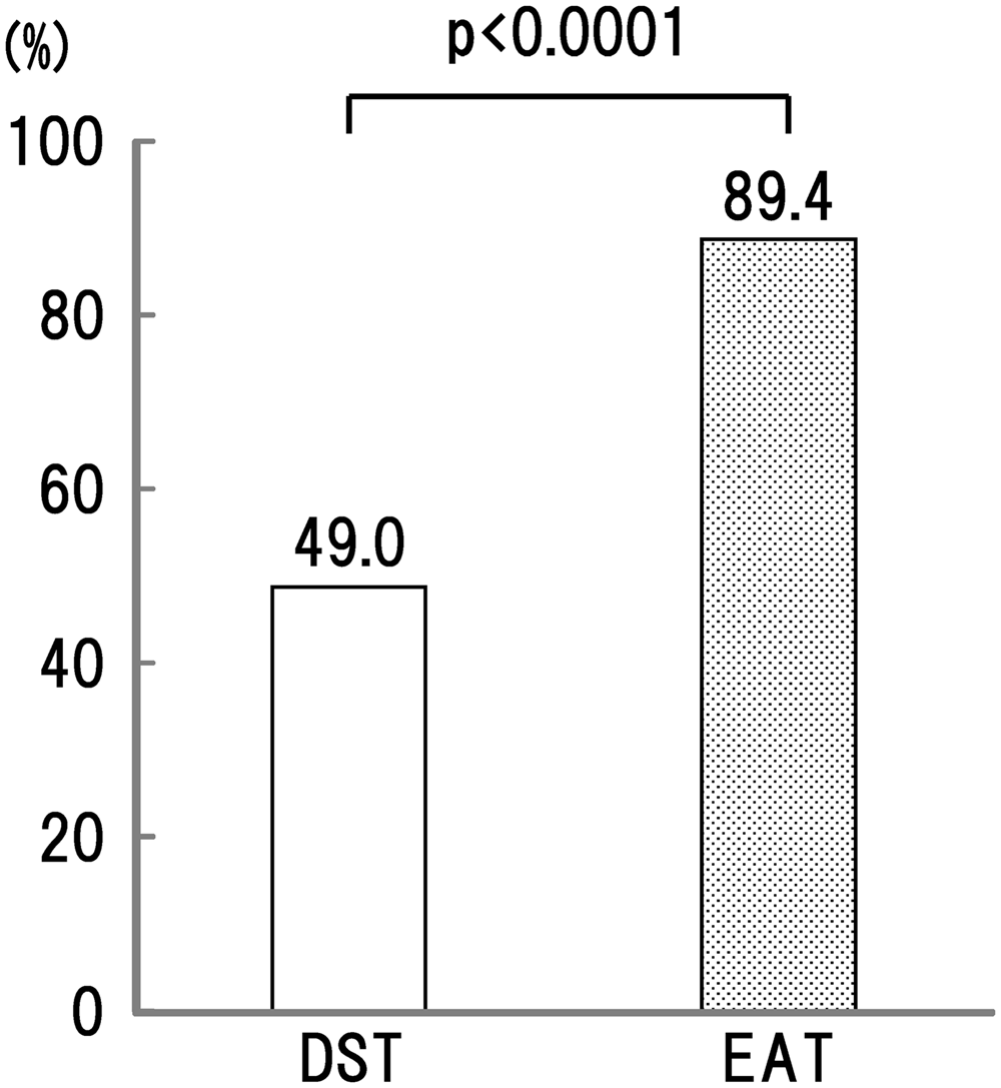
Achievement rates in each treatment group. The bars representing the DST group are white and the bars representing the EAT group are black.

Consistent with the larger reduction in LDL-C, the EAT treatment led to the achievement of the LDL-C targets in a significantly larger number of patients. When the patients with and without CVD were combined, 89.3% of the patients in the EAT group attained the optimized goal versus 51.0% of the patients in the DST group (Fig 5).

### Basic lipid chemistry

The analyses of basic lipid chemistry revealed a significantly larger percent change in TC in the EAT group than in the DST group (Fig 4D), whereas no differences were observed between the therapies in HDL-C or TG (Fig 4E and 4F). The reductions of actual LDL-C and apo B values were significantly larger in EAT than in DST (Figs 2B and 4C).

### Adverse events

Three cases of adverse events were reported before 12 weeks in the EAT group: a common cold, hepatocellular carcinoma, and eczema. None of them were considered to be related to the lipid treatment. The patient with eczema continued the medications until 12 weeks and then stopped them. The patient who developed hepatocellular carcinoma continued to take drugs.

We observed no adverse changes in HbA1c (from 7.24±0.65% to 7.34±0.80% in EAT; from 7.26±0.97% to 7.37±1.03% in DST). HOMA-IR, calculated for the patients with IRI measurement, revealed to be not different between therapies. With respect to liver function, we found no exacerbation. No overt complaints concerning muscles were recognized either group. A slight elevation of CPK was observed in the EAT patients, but not outside of the normal range.

## Discussion

This is the first prospective, randomized multi-center controlled trial to investigate the effect of ezetimibe add-on therapy on serum LDL-C concentrations compared to the intensified high-potency statin therapy (20 mg of atorvastatin or 2 mg of pitavastatin) in an Asian population of type 2 diabetics in whom high-potency statin (10 mg of atorvastatin or 1 mg of pitavastatin) had failed to bring LDL-C to the target levels. Our result indicated that ezetimibe add-on therapy brought about not only a significantly larger LDL-C decrement as the primary end point of this study, but also improved the atherogenic lipid profile as a secondary end point (by bringing about significantly larger sd-LDL-C and RLP-C decrements) compared to high-potency statin therapy (20 mg of atorvastatin or 2 mg of pitavastatin).

The percent reduction of LDL-C with EAT exceeded the percent reduction with DST by 13.7% in this study. This reduction was consistent with earlier studies on Caucasian diabetics [2], on type 2 diabetics receiving TZD monotherapy [23], and on type 2 diabetics with high CAD risk [24]. In a post-hoc analysis, ezetimibe-add-on therapy was equally effective for subjects with metabolic syndrome [25]. Our results more generally support the validity of ezetimibe for diabetic patients with a wide range of clinical backgrounds such as diabetes-medication, cardiovascular risk, and metabolic status.

Small LDL particles have several features that make them more atherogenic [26–28]. EAT treatment improved the typical atherogenic profile of diabetes by altering the LDL particle size and reduced the sd-LDL-C level more effectively than the DST treatment (20.5% vs. 3.7%). The RESEARCH study is also investigating the atherogenic lipid profile of RLP-C, an independent risk factor of CAD [29], and future coronary events in patients with CAD and type 2 diabetes [10, 30]. EAT decreased RLP-C by 19.7% in this study, while the DST increased it. The action of ezetimibe in the intestine may be a common mechanism underlying the changes of these two atherogenic lipoproteins. Ezetimibe is reported to reduce the cholesterol content in chylomicrons with apoB48, initiating a process that presumably reduces the cholesterol content of large VLDL and in turn decreases VLDL remnants and sd-LDL-C [31]. RLP-C levels are closely associated with CAD risk even in patients with normal triglyceride levels [32]. The plasma TG concentration after 12 weeks of ezetimibe add-on therapy was unchanged from baseline. The major component in RLP was VLDL remnant (IDL) because the samples measured in this study were taken during fasting. In recent experiments with pigs, the co-administration of simvastatin and ezetimibe decreased VLDL and LDL apoB-100 concentrations by reducing VLDL production and upregulating LDL receptor-mediated LDL clearance [33]. This finding attests to the potential of combination therapy to improve the lipid profile over a broad spectrum.

The target values defined in this study were taken from Japanese guidelines [17] for lipid control in diabetics based on two sources: first, evaluations of absolute risk that estimated a 10-year cardiovascular mortality of more than about 2% for diabetics [34]; second, a population-based study that estimated a 20% incidence of atherosclerotic disease in diabetics over 10 years [35]. Thus, the target values advocated by the Japanese guideline seem comparable to the NCEP guideline. The general rates of goal-achievement for conventional statin therapies are reported at around 50% even when the statin doses are doubled [36]. The rates fall further in patients in whom the first-line doses fail to achieve the LDL-C targets. Our achievement rates, 89.4% in EAT and 51.0% in DST, were higher than the rates from other studies investigating the effects of high-potency statin on patients in whom the first-line treatments failed to achieve the targets [37].

The subjects receiving the ezetimibe add-on therapy showed significantly larger reductions in non-HDL-C and ApoB, but not in TG or HDL-C. Among these basic lipids, the NCEP guidelines present target values for non-HDL-C. Our subjects receiving the EAT showed an additional 12.8% reduction in the absolute value of non-HDL-cholesterol, a change that may correspond to a greater than 10% reduction in cardiovascular risk [38]. This finding marks non-HDL-cholesterol as a potentially useful index for evaluating the effects of the treatment modification after the first-line statin therapies fail. Non-HDL-cholesterol and ApoB have been described as more useful markers for the prediction of cardiovascular disease [39]. Non-HDL-C appeared to be a more exquisite index for the ezetimibe treatment in our study, as the EAT brought about larger changes in non-HDL-C than in ApoB. As statin use grows more prevalent the world over, the position of these indices in the evaluation of residual risks should be further elucidated. The latest 2013 ACC / AHA Risk Assessment and Cholesterol Treatment Guideline [40] abandoned the target values of LDL-C. This evokes controversy as to the preferred treatment strategy for T2DM: Is it better to aim for target values or simply to administer moderate- or high- intensity statin [41, 42]? The Canadian Cardiovascular Society has issued an opposing view on this point [43]. While this new guideline attempts to define the population most favored by statin treatment by reviewing RCTs, ethnicity differences in risk evaluation and drug effects are overlooked. This makes it necessary to evaluate the adequacy of statin therapy by measuring LDL-C. Indeed, a more recent International Atherosclerosis Society Position Paper has set LDL-C and non-HDL-C targets for therapy and defined the optimal target LDL-C value as < 100 mg/dl [44]. The problem of residual risks left by statin therapy must also be addressed. In our data, the levels of sd-LDL and RLP-C correlated significantly with the level of non-HDL-C (data not shown). This aspect could contribute to a more precise risk evaluation and enhance the optimum care for individual T2DM patients.

The overall risk reduction by ezetimibe remains controversial. Ezetimibe has been shown to be effective for reducing cardiovascular events in two large-scale clinical trials: myocardial infarction was reduced in the SEAS study [45, 46], and patients receiving ezetimibe plus simvastatin suffered fewer major atherosclerotic events in the SHARP study [47]. In both studies, the degree of event reduction based upon the percent change in LDL-C was similar to what was previously observed in a meta-analysis [48]. Yet in another two trials, a high-potency statin monotherapy with pronounced lipid-lowering effects failed to significantly reduce major cardiovascular events in subjects with renal disease under dialysis [49].

An important limitation of this study should be pointed out. Regarding our endpoints, the study exhibited clear results. This may have stemmed from the mainly Asian makeup of our patient population. Indeed, this study design was bound by local regulations or recommendations including statin doses. The relatively low statin doses routinely administered to Asians including Japanese compared to Caucasians could have elicited more significant results. From this perspective, great care would be required to extrapolate our results to a worldwide recommendation. Modifications according to the actual local positions of statins would also be advisable.

In summary, a substantial number of patients receiving the add-on therapy with ezetimibe achieved target LDL-C levels determined to be optimal for reducing absolute risk by a significantly larger decrement in a population of subjects in whom first-line statin treatment failed. The ezetimibe therapy also modified several atherogenic lipoproteins, including sd-LDL and RLP-C, by improving the lipid profiles (particle sizes). These effects, in turn, may reduce the incidence of future atherogenic events. Our results prove that add-on ezetimibe is a suitable treatment for the persistent dyslipidemia in type 2 diabetes patients. This study may stand as an important landmark in the treatment of dyslipidemia for diabetic patients.

## Supporting Information

S1 CONSORT Checklist CONSORT Checklist. (DOC)

S1 Protocol Study protocol in Japanese (DOC)

S2 Protocol Study protocol translated into English (DOCX)

S1 Table Dataset of RESEARCH. (XLSX)
