# Supplementary material for: Effect of Ezetimibe on LDL-C Lowering and Atherogenic Lipoprotein Profiles in Type 2 Diabetic Patients Poorly Controlled by Statins
Source: PLoS One. 2015 Sep 23;10(9):e0138332. doi: 10.1371/journal.pone.0138332 (PMC4580589; doi:10.1371/journal.pone.0138332)
Supplement: S1 Protocol — (DOC) [file pone.0138332.s002.doc]

**自主臨床試験実施計画書**

自主臨床試験課題名：

「２型糖尿病患者に対するエゼチミブ/スタチン併用療法の有効性比較臨床試験」

研究代表者：柴　輝男

所属機関･診療科：東邦大学医療センター大橋病院　糖尿病・代謝内科

臨床試験実施予定期間：2009年7月1日～2013年6月30日

2009年 6月 4日 初版　作成

2010年4月12日　改訂版　　　　　　　　　　　　　　　　　　　　　　　　　　　　　2010年10月22日 改訂第2版

2011年10月31日 改訂第3版

2012年10月17日 改訂第4版

目次

1. 試験の背景……………………………………………………………………………………3
2. 試験の目的と必要性…………………………………………………………………………3
3. 試験薬の概要…………………………………………………………………………………4
4. 対象患者………………………………………………………………………………………4
5. 被験者に説明し同意を得る方法……………………………………………………………4
6. 試験の方法……………………………………………………………………………………5
7. 評価項目………………………………………………………………………………………6
8. 観察および検査項目……………………………………………………………… …………8
9. 中止基準……………………………………………… ………………………………………9
10. 有害事象発生時の取扱い……………………………………………………………………10
11. 実施計画書からの逸脱の報告………………………………………………………………10
12. 試験の終了、中止、中断……………………………………………………………………10
13. 試験実施期間…………………………………………………………………………………11
14. データの集計および統計解析方法…………………………………………………………11
15. 目標症例数……………………………………………………………………………………11
16. 被験者の人権および安全性・不利益に対する配慮………………………………………12
17. 患者の費用負担………………………………………………………………………………12
18. 健康被害の補償および保険への加入………………………………………………………12
19. GCPおよびヘルシンキ宣言への対応………………………………………………………12
20. 記録の保存……………………………………………………………………………………12
21. 研究結果の公表………………………………………………………………………………12

自主臨床試験：「２型糖尿病患者に対するエゼチミブ/スタチン併用療法の有効性比較臨床試験」

**試験実施計画書**

**1．試験の背景**

生活習慣の欧米化に伴い、糖尿病をはじめとする生活習慣病が大きな問題となってきている。本邦における糖尿病患者は年々増加傾向にあり、厚生労働省が発表した「平成18年国民健康・栄養調査」によると「糖尿病が強く疑われる人」は約820万人、「糖尿病の可能性が否定できない人」は約1,050万人であり、糖尿病の該当者かその予備軍と推定された人の合計は約1,870万人に上り、平成14年の調査より250万人（15.4%）増加している。また、２型糖尿病患者の増加と共に、合併症疾患が増加しており、特に虚血性心疾患、脳血管障害など大血管症の発症率が増加しており、血糖管理に加えて、脂質代謝異常や高血圧の是正の重要性が明らかになっている。

　以前より、スタチンによる心血管イベントの抑制効果については多くの報告がなされている。しかし、2004年に発表されたJapan Diabetes Complications Study (JDCS)の第６年次中間報告にあるように、虚血性心疾患発症率は6.7/1000人/年、脳血管障害発症率は6.5/1000人/年であり、久山町研究の非糖尿病患者のイベント発症率と比較すると、３～４倍の高値を示しており、現在でも、２型糖尿病患者における心血管イベント発症は、大きな問題となっている。

動脈硬化の主要なリスクファクターであるLDLコレステロールには2つの供給源があることが知られている。1つは肝臓における合成であり、もう1つは小腸からの吸収である。現在の脂質改善療法については、肝臓におけるコレステロール合成抑制を治療ターゲットとしたスタチンが主流であり、２型糖尿病患者を対象にスタチンによるイベント抑制および様々な炎症マーカーを低下させることは数多く報告されている。

もう1つの治療ターゲットであるコレステロール吸収についても近年、小腸近位部に発現するコレステロールトランスポーターNPC1L1の分子生物学的および臨床研究の発展により、コレステロール吸収抑制の臨床的意義が明らかにされつつある。更に、昨年新たにNPC1L1に結合することによりコレステロール吸収を抑制するエゼチミブの使用が可能になった。また、海外の臨床研究によるとスタチンの増量より本剤との併用のほうが脂質低下が強力であるとの報告がなされている。

しかし、国内におけるコレステロール吸収抑制剤（エゼチミブ）とスタチン併用による臨床効果については十分な報告が無いのが現状である。

**2．試験の目的と必要性**

1. 目的

２型糖尿病を合併する高コレステロール血症に対するエゼチミブ/スタチン併用療法の治療効果について検討する。同時に両群における炎症マーカーを測定することにより抗炎症作用を明らかにする。

1. 本試験の必要性・臨床的意義

2型糖尿病患者への有効な高コレステロール治療を見出すことにより、より有用な治療を確立できる可能性があり、また、心血管イベントの予測因子とされる炎症マーカーへの影響も明らかにすることにより、更なる治療効果の向上に繋がる方法を見出す可能性がある。

**3．試験薬の概要**

1. アトルバスタチンカルシウム錠

商品名：リピトール錠5mg、10mg

一般名：アトルバスタチンカルシウム水和物錠

1. ピタバスタチンカルシウム製剤

商品名：リバロ錠1mg、2mg

一般名：ピタバスタチンカルシウム製剤

1. エゼチミブ

商品名：ゼチーア錠10mg

一般名：エゼチミブ錠

**4．対象患者**

以下の選択基準を満たし、除外基準に抵触しない患者

1. 選択基準

### 2型糖尿病患者

### アトルバスタチン10mgまたはピタバスタチン1mgを1ヶ月以上投与しかつLDL-Cが120mg/dL以上の患者（冠動脈疾患の既往のある患者は100mg/dl以上）

### 年齢は20歳以上

1. 除外基準

### 試験薬に過敏症の既往歴のある患者

### トリグリセライド値が400mg/dLを超える患者

### 肝機能障害を有する患者（ALT＞2×施設正常上限値）

### コントロール不良な糖尿病患者（HbA1cが9%以上）

### 腎機能障害を有する患者（血清クレアチニン＞2.0mg/dL）

### 二次性高脂血症患者および薬剤性高脂血症患者

### ホモ接合型家族性高コレステロール血症患者

### 妊娠中・授乳中の患者または妊娠している可能性のある患者

### その他、主治医が本検討に際し、参加が不適当であると判断した患者

**5．被験者に説明し同意を得る方法**

担当医師は本学の治験審査委員会で承認の得られた同意説明文書を患者に渡し、文書および口頭による十分な説明を行い、患者の自由意思による同意を文書で得る。

患者の同意に影響を及ぼすと考えられる有効性や安全性等の情報が得られたときや、患者の同意に影響を及ぼすような実施計画等の変更が行われるときは、速やかに患者に情報提供し、試験等に参加するか否かについて患者の意思を予め確認するとともに、事前に治験審査委員会の承認を得て同意説明文書等の改訂を行い、患者の再同意を得る。

**6．試験の方法**

1. 試験の種類・デザイン

多施設、非盲検、実薬対照、最小化法、並行群間比較試験

1. 試験のアウトライン

アトルバスタチン20mg又はピタバスタチン2mg

同意取得

登録・最小化法割付

アトルバスタチン10mg又はピタバスタチン1mg +エゼチミブ10mg

投与期間　（12週間）*

*：但し、追跡可能な患者においては1年間継続投与を行い、長期有効性・安全性を確認する。

12週後、LDL-Cが目標値に達していない患者には主治医の判断で使用中のスタチンの増量またはエゼチミブの追加投与も可能とする。

1. 被験者の試験参加予定期間

全期間：12週間（但し、追跡可能な患者においては1年間）

投薬期間：12週間（但し、追跡可能な患者においては1年間）

1. 試験薬の用法・用量、投与期間
   1. 試験開始から12週間、割り付けられた試験薬を以下の用量で1日1回食後に服用する。

・アトルバスタチンとエゼチミブ併用群：アトルバスタチン10mg錠1錠、エゼチミブ10mg錠1錠

・ピタバスタチンとエゼチミブ併用群：ピタバスタチン1mg錠1錠、エゼチミブ10mg錠1錠

・アトルバスタチン単独投与群：アトルバスタチン10mg錠2錠

・ピタバスタチン単独投与群：ピタバスタチン2mg錠1錠または1mg錠2錠

試験期間を通じて一定の時刻に服用する。

午前中に服用する場合、来院日は検査後に服用する。

- 1. 設定の根拠

それぞれの薬剤の添付文書に記載の用法・用量に従って設定した。なお、エゼチミブは添付文書において「通常、成人にはエゼチミブとして1回10mgを1日1回食後経口投与する。」とされているため、食後投与と設定した。

1. 併用薬（療法）に関する規定
   1. 併用禁止薬
2. 以下の血清脂質低下薬（本試験で投与する薬剤を除く）

HMG-CoA還元酵素阻害薬、陰イオン交換樹脂製剤（コレバイン），フィブラート製剤（リピディル、リパンチル、ベザトールなど）、ニコチン酸製剤（ペリシッド）、EPA製剤（エパデール）、プロブコール(シンレスタール、ロレルコなど)、その他の血清脂質低下薬。

試験薬の有効性・安全性を正確に評価するため設定した。

1. シクロスポリン

シクロスポリンはピタバスタチンと併用禁忌であるため。また、シクロスポリンとアトルバスタチンおよびエゼチミブとの併用により両薬剤の血中濃度の上昇が認められているため、副作用発現リスクが高いと考えられるため。

- 1. 併用可能薬（療法）

合併症および有害事象に対する治療薬は適宜使用してもよい。但し、使用した薬剤についてはその薬剤群を調査票に記載する。

1. 症例登録、割付方法

試験責任（分担）医師は患者が選択基準を満たし除外基準に抵触しないことを確認した後、①本研究参加への患者の自由意思による同意を文書にて取得する。②試験責任（分担）医師は「登録用紙」に、被験者と被験者識別番号を対応させるために必要な事項を記載する。③Webに必要項目を入力し、被験者識別番号の確定と割付を行う。④試験責任（分担）医師は、「登録用紙」にWebにて表示された、被験者識別番号と割付結果を記載し保存する。

**7．評価項目**

1. 主要評価項目

開始時のLDLコレステロール値を基準とした試験薬投与終了時のLDL-コレステロール変化率（%）

[設定根拠]

「抗高脂血症薬の臨床評価方法に関するガイドライン（1988）」では、脂質の変化率を指標とすることが評価方法の一つとして示されており、LDL-コレステロール変化率は有効性の評価方法として国内外で用いられている。

1. 副次的評価項目

終了時にLDL-コレステロールが脂質目標管理値に到達した割合

開始時の値を基準とした試験終了時の血清脂質および炎症マーカーの変化率（%）

＊解析時にはインスリン投与患者のインスリン値は使用しない。ただし、二次解析時には使用する可能性もある。

[設定根拠]

「動脈硬化性疾患予防ガイドライン（2007年版）」において、日本人の脂質管理目標が危険因子の有無により分類したカテゴリーごとに示されている。したがって、試験終了時のLDL-コレステロール値が目標管理値に到達した被験者の割合は、有効性の指標として考えられた。

LDL-コレステロール以外にも動脈硬化の危険因子として考えられる血清脂質があり、血清脂質への影響を評価することは有効性の指標として妥当と考えられた。また、動脈硬化疾患の予測因子として、炎症マーカーが有効であると報告されていることから、炎症マーカーへの影響を評価することは有効性の指標として妥当と考えた。

1. 安全性評価

副次評価項目

有害事象および副作用

臨床検査値

- - - 1. 有害事象の定義

有害事象とは医薬品が投与された際に起こる、あらゆる好ましくない、あるいは意図しない徴候（臨床検査値異常を含む）、症状、病気のことであり、当該試験薬との因果関係の有無は問わない。有害事象のうち試験薬との因果関係が否定できないものを副作用という。

- - - 1. 症状の程度

発熱以外の有害事象の程度は以下の基準に従って判定する。

① 軽度（軽微である） ：日常生活に支障を及ぼさない程度

② 中等度（重篤でない、軽微でない）：日常生活に制限を受ける程度

③ 高度（重篤である） ：日常生活を遂行できない程度

発熱に関しては以下の基準に従って判定する。

① 軽度 ：38℃未満でかつ，投与前と比べ明らかに発熱していると思われる状態

② 中等度 ：38℃以上40℃未満

③ 高度 ：40℃以上

- - - 1. 重篤な有害事象の定義

有害事象が下記のいずれかに該当する場合、重篤な有害事象として取り扱う。

① 死亡

② 死亡につながる恐れのある事象

③ 入院または入院期間の延長が必要とされる事象

④ 障害（永続的または顕著な障害・機能不全に陥るもの）

⑤ 先天異常を来たすものまたは出生異常

⑥ 上記に準じて重篤である事象（その他の医学的に重大な状態）

- - - 1. 処置

処置について下記のように分類する。

① 継続　② 減量　③ 中止

- - - 1. 有害事象の転帰

下記のように分類・判定し，症例報告書に記入する。

① 回復（症状が消失した場合）

② 軽快（症状の重症度が低くなった場合）

③ 未回復

④ 後遺症あり（回復したが、被験者の通常の生活が妨げられる場合、例えば継続的な薬物治療が必要なもの等）

⑤ 死亡（死亡の原因が当該有害事象による場合）

⑥ その他（その理由も明記する）

- - - 1. 因果関係

試験薬との因果関係について下記の区分で判定する。

① 関連なし　② ほとんどない　③ 否定できない　④ 関連あり

**8．観察および検査項目**

1. 患者背景

1) 一般的項目 ：性別、年齢、身長、体重、既往歴（脳・心血管系のみ）、合併症、喫煙の有無、冠動脈疾患の家族歴、併用薬（薬剤群）

1. 検査項目

1) 血清脂質関連 ：総コレステロール（TC）、トリグリセライド（TG）、LDL-コレステロール（LDL-C）、HDL-コレステロール（HDL-C）、RLP-C、apo A-1、apo B、apo E（開始時、12週後、26週後、52週後）

MDA-LDL（CHD既往の患者のみ）（開始時、12週後、26週後、52週後）

small dense LDL（開始時、12週後、52週後）

2) 糖代謝　　　　：空腹時血糖値、インスリン、HbA1c（開始時、12週後、26週後、52週後）

3) 炎症マーカー　：hs-CRP（開始時、12週後、52週後）

4) 血液生化学検査：ALT、AST、γ-GTP、CPK、血清クレアチニン

（開始時、12週後、26週後、52週後）

検査スケジュール

|  | | 観察期 | 開始時 | 12週後 | 26週後* | 52週後* |
| --- | --- | --- | --- | --- | --- | --- |
| 来院のずれの許容範囲 | |  |  | +4週 | ±4週 | ±4週 |
| 同意取得 | | **●** |  |  |  |  |
| 患者背景 | | **●** | |  |  |  |
| 血清脂質関連 | TC |  | **●** | **●** | **●** | **●** |
|  | TG |  | **●** | **●** | **●** | **●** |
|  | LDL-C |  | **●** | **●** | **●** | **●** |
|  | HDL-C |  | **●** | **●** | **●** | **●** |
|  | RLP-C |  | **●** | **●** | **●** | **●** |
|  | Apo A-1 |  | **●** | **●** | **●** | **●** |
|  | Apo B |  | **●** | **●** | **●** | **●** |
|  | Apo E |  | **●** | **●** | **●** | **●** |
|  | MDA-LDL** |  | **●** | **●** | **●** | **●** |
|  | small dense LDL |  | **●** | **●** |  | **●** |
| 糖代謝 | 空腹時血糖 |  | **●** | **●** | **●** | **●** |
|  | **インスリン^＃^** |  | **●** | **●** | **●** | **●** |
|  | HbA1c |  | **●** | **●** | **●** | **●** |
| 炎症マーカー | hs-CRP |  | **●** | **●** |  | **●** |
| 生化学検査 | ALT、AST、γ-GTP、CPK、血清クレアチニン |  | **●** | **●** | **●** | **●** |
| 有害事象 | |  | **●** | | | |

●：必須検査、*：実施可能な患者のみ、**：CHD既往の患者のみ

＃:インスリン投与患者における測定は担当医師の判断

**9．中止基準**

本試験中の症例において、以下のいずれかに該当し、試験責任（分担）医師が必要と認めた場合、被験者への投与を中止し、安全を確保するとともに適切な処置を行う。

1. 被験者から試験参加の辞退の申し出や同意の撤回があった場合
2. 登録後に適格性を満足しないことが判明した場合
3. 転居、転院または来院しなくなった場合
4. 原疾患の悪化、不慮の事故または有害事象の発現により試験薬の投与継続が好ましくないと判断された場合
5. その他の理由により、医師が試験を中止することが適当と判断した場合

**10．有害事象発生時の取扱い**

- 1. 有害事象発生時の被験者への対応

試験責任（分担）医師は、有害事象を認めたときは、直ちに適切な処置を行うとともに、カルテならびに症例報告書に齟齬なく記載する。また、試験薬の投与を中止した場合や、有害事象に対する治療が必要となった場合には、被験者にその旨を伝える。

- 1. 重篤な有害事象の報告

試験責任医師は、重篤な有害事象の発生を認めたときは、速やかに病院長に報告する。

重篤な有害事象の定義

1) 死亡に到るもの

2）生命を脅かすもの（死亡のリスクにさらすような事象）

3) 治療のための入院または入院・加療期間の延長が必要なもの

4）永続的もしくは顕著な障害・機能不全に陥るもの

5）先天異常を来すものまたは出生異常

6）その他重大な医学的事象*

*即座に生命を脅かしたり、死亡や入院に到らなくても、上記のような結果に至らぬように処置を必要とするような医学的に重要な事象の場合には、重篤な有害事象とみなされる場合がある。

例として、救急治療室または自宅での集中治療を必要とするアレルギー性気管支炎、入院には至らない血液疾患または麻痺などがある。

試験期間中の全ての重篤な有害事象、試験終了（中止）後に試験薬との関連性が疑われる重篤な有害事象について報告する。

**11．実施計画書からの逸脱の報告**

1. 試験責任（分担）医師は、研究代表者の事前の合意および治験審査委員会の事前の審査に基づく病院長の承認を得る前に、試験実施計画書からの逸脱あるいは変更を行わない。
2. 試験責任（分担）医師は、緊急回避等のやむを得ない理由により、研究代表者との事前の合意および治験審査委員会の事前の承認を得る前に、試験実施計画書からの逸脱あるいは変更を行うことができる。その際には、試験責任（分担）医師は、逸脱または変更の内容および理由ならびに試験実施計画書等の改訂が必要であればその案を速やかに、研究代表者および治験審査委員会に提出し、研究代表者、治験審査委員会および病院長の承認を得る。
3. 試験責任（分担）医師は、試験実施計画書からの逸脱があった場合は、逸脱事項をその理由とともに全て記録し、試験責任医師は、医療機関で定めた所定の様式により病院長および試験の代表者に報告しなければならない。試験責任医師は、これらの写しを保存する。

**12．試験の終了、中止、中断**

(1) 試験の終了

試験責任医師は試験の終了時には速やかに試験終了報告書を病院長に提出する。

(2) 試験の中止、中断

試験責任医師は、以下の事項に該当する場合は試験実施継続の可否を検討し、試験の中止または中断を決定した時は、速やかに病院長に文書で報告する。

1) 試験薬の品質、安全性、有効性に関する重大な情報が得られたとき。

2) 被験者のリクルートが困難で予定症例を達成することが到底困難であると判断されたとき。

3) IRBにより、実施計画等の変更の指示があり、これを受入れることが困難と判断されたとき。

**13．試験実施期間**

平成 21年 7 月 1 日から平成 25 年 6 月 30 日（登録締切 24 年 6 月 30 日）

**14．データの集計および統計解析方法**

1. データの収集・集計方法

本試験用に作成された調査票に各施設の試験分担医師が記載し、事務局に送付する。事務局は収集したデータをExcelファイルに入力し集計する。

1. 解析対象集団の定義

本試験データについて、下記の定義のように解析、評価を行うこととする。

| 解析対象集団 | 定義 | 解析 |
| --- | --- | --- |
| 最大の解析対象集団  有効性のITT（Intention To Treat） | 同意取得後、登録された全例。ただし投与が1回もされない症例は除く。 | 背景、有効性、  安全性 |
| 実施計画書に適合した集団  有効性のPPS（Per Protocol Set） | 最低限の試験規定を完了しており、主要変数の測定値が利用可能な状態で、有効性に関わる重大な試験実施計画書違反がない症例 | 背景、有効性、  安全性 |
| 安全性解析対象集団 | 投与が行われ、投与後の安全性の評価が一つでも行われている全例 | 背景、安全性 |

1. 統計解析方法
   1. 有効性の解析

割付された群別の治療終了後12週時の治療期間開始時に対する変化率（%）についてWilcoxon rank sum testを行う。

治療終了時について、測定値が管理目標値に到達した被験者の割合を算出する。

群内の検査値の推移については、Wilcoxon signed rank testを行う。

- 1. 安全性の解析

本研究の適格例、不適格例に関わらず、一度でも試験薬投与が行われた症例に対して有害事象の発現例数および発現頻度を算出する。また、副作用についても同様の集計を行う。

**15．目標症例数および設定根拠**

1. 目標症例数

試験全体 ： 参加施設数 　25施設、目標症例数 120例

1. 例数設定の根拠

スタチン初回用量からのLDL-C低下効果をスタチン倍量投与群において11.7%、ゼチーア併用群において21.7%、SDを15%と仮定する。

αを0.05、Powerを90%、ドロップアウトを20%とすると60例/群となる。

**16．被験者の人権および安全性・不利益に対する配慮**

(1) 人権への配慮（プライバシーの保護）

試験実施に係る生データ類および同意書等を取扱う際は、被験者の秘密保護に十分配慮する。病院外に提出する症例報告書等では、被験者識別コード等を用いて行う。試験の結果を公表する際は、被験者を特定できる情報を含まないようにする。試験の目的以外に、試験で得られた被験者のデータを使用しないものとする。

(2) 安全性・不利益への配慮

有害事象発生時には速やかに適切な診察と処置を行う。

**17．患者の費用負担**

試験薬は保険診療範囲内で投与される。患者は健康保険診察の自己負担分を負担する。原疾患に対する検査、診察にかかわる費用は保険診療によるものとする。ただし、LDL-C、RLP-C、small dense LDL、hs-CRPの測定については研究費で実施するため、本試験に参加することで患者の費用負担が増えることはない。

**18．健康被害の補償および保険への加入**

1. 健康被害の補償

試験への参加に起因して生じた健康被害を本院にて治療した場合の治療費のうち、患者負担分について医療費減免制度に基づく申請を行う。ただし、以下の項目に関しては医療費減免制度を適応しない。

1. 患者の原疾患および合併症の治療に通常行われる診療の費用
2. 市販の薬剤を適応内で使用したことによる健康被害の治療費
3. 医療過誤等により賠償責任が問われる場合の治療費
4. 賠償保険への加入

賠償責任に備え、試験責任医師および試験分担医師は賠償責任保険に加入する。

**19．GCPおよびヘルシンキ宣言への対応**

本研究は「ヘルシンキ宣言」（2004年注釈追加）ならびに「臨床研究に関する倫理指針」（平成１６年厚生労働省告示第459号）を遵守する。

**20．記録の保存**

試験責任医師は、試験等の実施に係わる必須文書（申請書類の控え、病院長からの通知文書、各種申請書・報告書の控、登録用紙、同意書の控え、症例報告書、その他データの信頼性を保証するのに必要な書類または記録など）は試験責任医師が保管し、研究発表後5年後に廃棄する。

**21．研究結果の公表**

本試験の公表に関しては、試験終了後に試験責任医師、試験分担医師が相談し、公表時期、発表者、発表方法等を決定する。
