# Supplementary material for: Effect of Ezetimibe on LDL-C Lowering and Atherogenic Lipoprotein Profiles in Type 2 Diabetic Patients Poorly Controlled by Statins
Source: PLoS One. 2015 Sep 23;10(9):e0138332. doi: 10.1371/journal.pone.0138332 (PMC4580589; doi:10.1371/journal.pone.0138332)
Supplement: S2 Protocol — (DOCX) [file pone.0138332.s003.docx]

Implementation plan for a physician-led clinical trial

Name of Clinical Trial:

Recognized Effects of Statin and Ezetimibe Therapy towards Reaching the LDL-C Goal

Scheduled period for the clinical trial: from July 1, 2009 to June 30, 2013

First edition created June 4, 2009

Revised edition October 17, 2012

Table of Contents

1. Background leading up to this study
2. Purpose and needs of the study
3. Brief overview of the study drugs
4. Study patients
5. Policy and procedure for explaining the study and obtaining consent
6. Trial methods
7. Endpoints
8. Observation and examination
9. Criteria for terminating treatment
10. Handling of adverse events
11. Reporting on deviations from the protocol
12. Termination, cessation, or suspension of the trial
13. Study period
14. Data collection and statistical analysis
15. Target number of cases and basis for setting it
16. Safety, human rights, and disadvantages faced by the study subjects
17. Cost burdens on the patients
18. Insurance coverage and compensation for health problems resulting from the study
19. Observance of the Declaration of Helsinki and GCP
20. Storage of records
21. Publication of research results
22. Background leading up to this study

With the ongoing westernization of many facets of Japanese lifestyle, diabetes and other lifestyle-related diseases have become a major problem for Japan. The diabetic population in Japan is increasing year by year. According to the 2006 National Health and Nutrition Survey conducted by the Ministry of Health, Labor and Welfare, 10.5 million Japanese people are confirmed diabetics and another 8.2 million are strongly suspected to have the disease. Hence, 18.7 million people are likely to be diagnosed with diabetes in the future, or 2.5 million more than were recorded a few years earlier in the survey of 2002 (an increase of 15.4%). With this increase in the diabetic population comes a higher incidence of diabetic microangiopathies and associated forms of ischemic heart disease and cerebrovascular disease. Improvements not only in glycemic control, but also in blood pressure and dyslipidemia, are recognized as important preconditions for good prognosis in these populations.

Many reports have presented the inhibitory effects of statins on cardiovascular events. Yet according to the sixth annual interim report on the Japan Diabetes Complications Study (JDCS) published in 2004, the incidence rates of ischemic heart disease and cerebrovascular disease in diabetic populations were 6.7 / 1,000 humans per year and 6.5 / 1000 humans per year, or 3-4 times higher than the rates in the non-diabetic patients reported in the Hisayamacho study. Even now, the onset of cardiovascular events in patients with type 2 diabetes has become a major problem.

LDL cholesterol, a major risk factor for arteriosclerosis, comes from two sources: synthesis in the liver and absorption from the small intestine. Statins, the current first-line therapy for dyslipidemia, work mainly by inhibiting cholesterol synthesis in the liver. Statins have been reported to inhibit cardiovascular events and are confirmed to reduce various inflammatory markers in patients with type 2 diabetes. Recent progress in molecular biology has also made headway towards another clinical target, suppressing cholesterol absorption. Specifically, an inhibitor of the cholesterol transporter NPC1L1 expressed in the proximal portion of the small intestine has been found to inhibit cholesterol absorption in clinical studies. Ezetimibe, an agent that suppresses cholesterol absorption by binding to NPC1L1, has been accepted for clinical application in Japan since last year. According to clinical studies overseas, a combination of ezetimibe and statin is capable of conferring better control in the treatment of dyslipidemia compared to a stronger dose of statin.

Yet as of the present, there have yet to be appropriate reports in Japan on clinical results attained from the combination of the cholesterol absorption inhibitor ezetimibe with statins.

2. Purpose and needs of the study

1. Purpose

To evaluate the effect of combination therapy with ezetimibe and statin on type 2 diabetic subjects with hypercholesterolemia. The anti-inflammatory effects will be revealed by measuring inflammatory markers in both groups at the same time.

1. Needs of the study and clinical significance

The discovery of a more potent treatment for type 2 diabetics with hypercholesterolemia could lead to the establishment of a useful therapy with pleiotropic effects for type 2 diabetics overall. It may also be possible to further improve the therapeutic effects on type 2 diabetics by understanding how the combination treatment of ezetimibe and statin influences inflammatory markers predictive of cardiovascular events.

1. 3. Brief overview of the study drugs
2. Atrovastatin calcium tablet

Trade name: Lipitor tablet 5 mg and 10 mg

Generic name: Atrovastatin calcium hydrate

1. Pitavastatin calcium tablet

Trade name: Livalo tablet 1 mg and 2 mg

Generic name: Pitavastatin calcium tablet

1. Ezetimibe

Trade name: Zetia tablet 10 mg

Generic name: ezetimibe tablet

1. Study patients

Patients who meet the following entry criteria without meeting any of the following exclusion criteria

1. Entry criteria
2. Type 2 diabetes mellitus
3. Has used atorvastatin 10 mg/day or pitavastatin 1 mg/day for >1 month
4. LDL-C ≥120 mg/dL for patients without CAD; LDL-C ≥100 mg/dL for patients with CAD
5. Age ≥20 years

(2) Exclusion criteria

1. History of hypersensitivity to atorvastatin, pitavastatin, or ezetimibe
2. Triglyceride ≥400 mg/dL
3. Hepatic dysfunction (ALT> 2 x upper limit of normal range in each hospital)
4. Poor glycemic control (HbA1c > 9.4 %)
5. Renal dysfunction (serum creatinine ≥2.0 mg/dL)
6. Secondary dyslipidemia, drug-induced dyslipidemia
7. Homozygotic familial hypercholesterolemia
8. Pregnant, able to conceive children, or breastfeeding
9. Another non-specific reason, such as poor adherence

5. Policy and procedure for explaining the study and obtaining consent

Sub-investigators will give each patient a consent document with descriptions of the study approved by the Institutional Review Board of the University and thoroughly explain the content of the document to the patient verbally. After discussion and an exchange of questions and further explanations, the patient will provide his or her voluntary written consent to participate in the study.

When new information on safety or efficacy becomes available or the implementation plan, etc. is to be changed in any way that the investigators believe may influence the patients’ willingness to continue participating in the trial, the patients will be informed of the details and asked if they intend to continue participating. Those that express an intention to continue participating will be given a revised written description of the study approved by the Institutional Review Board and requested to once more give their consent.

6. Trial Methods

(1) Trial design

Multicenter, non-blind, active control, minimization method, parallel-group study

1. Research outline

Atorvastatin 20 mg or pitavastatin 2 mg

Registration and assignment by minimization method

Informed consent

Atorvastatin 10 mg or pitavastatin 1 mg + ezetimibe10mg

administration period（12 weeks）*

* The treatment would be modified in patients who fail to reach target levels during extended periods after 12 weeks under the clinical decision of each sub-investigator, and the effects of the modifications will be observed. Meanwhile, long-term effects will also be evaluated in subjects without treatment modification in each regimen group in the same period.

1. Scheduled study period

Total: 12 weeks (extended to 1 year if possible)

Administration period: 12 weeks (extended to 1 year if possible)

1. Dose regimen and administration period
   1. The subjects will take the assigned drugs postprandially once a day for 12 weeks from the starting point.

・Group receiving the combination of atorvastatin and ezetimibe: one 10 mg tablet of atorvastatin and one 10 mg tablet of ezetimibe daily

・Group receiving the combination of pitavastatin and ezetimibe: one 1 mg tablet of pitavastatin and one 10 mg tablet of ezetimibe daily

・Group receiving atorvastatin monotherapy: two 10 mg tablets of atorvastatin daily

・Group receiving pitavastatin monotherapy: one 2 mg tablet or two 1 mg tablets of pitavastatin daily

The patients will take the drugs at a fixed time each day during the study period.

On hospital visits, they will take the drugs after the laboratory examination even if they are normally scheduled to take the drugs in the morning.

- 1. Basis for dose setting

Doses were determined according to the product documentation. The subjects will take ezetimibe postprandially, as the product documentation for ezetimibe states: “Ezetimibe prescribed for adults is usually given once a day at a 10 mg dose postprandially.”

1. Rules for combination therapy
   1. Prohibited drug combinations
2. Lipid-lowering agents (except for the study drugs):

HMG-CoA reductase inhibitors, anion-exchange resins, fibrates, nicotinate, EPA, probucol, and other lipid-lowering agents are not to be taken in combination with the study drugs.

To evaluate the efficacy and safety of the study drugs precisely.

1. Cyclosporine

The combination of cyclosporine and pitavastatin is contraindicated. The combination of cyclosporine with atorvastatin or ezetimibe leads to high serum levels of these drugs, which increases the risk of side effects.

- 1. Drugs acceptable for combination therapy

Drugs for complications and adverse events can be used as prescribed. All drugs used must be described on the examination sheets.

1. Methods of registration and assignment

The sub-investigators will check to confirm that the patient meets the inclusion criteria and does not meet the exclusion criteria. Next, the doctors will (1) obtain the patient’s voluntary written consent to enroll in this study, (2) fill in the “registration sheet” to pair the subject with an identification number, (3) fix the identification number and allocate the patient to a regimen by uploading the necessary data to the web, (4) fill in the indicated identification number and result of the allocations on the “registration sheet,” and preserve the sheet.

7. Endpoints

(1) Primary endpoint

The primary endpoint is the percentage change in the level of serum LDL-C from baseline at the end of the administration period.

(Basis for setting the primary endpoint)

The Japanese guideline for clinical evaluation of lipid lowering agents in 1988 suggests the rate of lipid change as a standard index for evaluation, and the rate of change in LDL-cholesterol is widely used as an index for efficacy domestically and worldwide.

(2) Secondary endpoints

The first secondary endpoint is the percentage of patients who reach the treatment goal for LDL-C at the end of the administration period.

The second secondary endpoint is the percentage change in the levels of serum lipids and inflammation markers from baseline to the end of the administration period.

* The serum IRI values of the patients injecting insulin will not be analyzed initially, but they may be analyzed by a secondary analysis.

(basis of setting)

The Japanese “Guideline for the Prevention of Atherosclerotic Diseases (2007)” presents target lipid-lowering values for different categories classified by risk factors. Based on these values, the percentage of patients who reach the treatment goals for LDL-C at the end of the administration period will be adopted as an index of efficacy. As some serum lipids other than LDL-C are recognized as risk factors for atherosclerosis, the effects on these lipids will also be evaluated as appropriate indices of efficacy. Several inflammatory markers reported to be predictive of atherosclerosis will also be evaluated as appropriate indices of efficacy.

1. Evaluation of safety

Secondary endpoints

Adverse events and side effects

Laboratory examination

1. Definitions of adverse events and side effects

Adverse events mean any unfavorable or unintended signs (including abnormal laboratory data), symptoms, or diseases, regardless of causal associations with the study drugs. Side effects mean adverse effects that cannot be confirmed not to be causally associated with study drugs.

1. Severity of adverse events

The severity of adverse events other than fever is determined by the following criteria:

1. Slight : no trouble in daily living.
2. Intermediate (non-severe, non-slight): some restrictions in daily living
3. Severe: independent daily not possible

The severity of fever is determined by the following criteria:

1. Slight: body temperature of less than 38℃ but apparently higher than baseline
2. Intermediate: body temperature of more than 38℃ but less than 40℃
3. Severe: body temperature of more than 40℃
4. Definition of severe adverse events

The following will be treated as severe adverse events:

1. Death
2. Events that may lead to death
3. Hospital admission or extended period of hospitalization
4. Disability (permanent or remarkable disability/dysfunction)
5. Events that cause congenital anomalies or birth anomalies
6. Events that resemble any of the abovementioned states
7. Treatment adjustments for adverse events

The treatment adjustments for adverse events will be classified into three types:

1. Continuation 2. Dose reduction 3. Cessation
2. Outcome

The outcomes of adverse events will be described as follows on the case sheets:

1. Recovery (symptoms disappear)
2. Remission (severity of symptoms decreases)
3. Recovery delay
4. After-effect (daily living is still disrupted after recovery, requiring continuous medication)
5. Death (caused by relevant adverse events)
6. Other (clarify the reason)
7. Causative association

Causative associations will be classified as follows:

1. No relation 2. Little relation 3. Undeniable relation 4. Related

8. Observation and examination

(1) Patient background

1) general assessment: sex, age, height, body weight, past history (stroke or cardiovascular), complications, smoking, family history of cardiovascular disease, co-administered drugs

(2) examination component

1) serum lipids: total cholesterol (TC), triglyceride (TG), LDL-cholesterol (LDL-C), HDL-cholesterol (HDL-C), RLP-C, apo A-1, apo B, apo E (baseline, 12 wk, 26 wk, 52 wk); MDA-LDL (only for patients with CHD history) (baseline, 12 wk, 26 wk, 52 wk), small dense LDL (baseline, 12 wk, 52 wk)

2) glucose metabolism: fasting plasma glucose, IRI, HbA1c (baseline, 12 wk, 26 wk, 52 wk)

3) inflammatory markers: hs-CRP (baseline, 12 wk, 52 wk)

4) biochemical: ALT, AST, γ-GTP, CPK, serum creatinine (baseline, 12 wk, 26 wk, 52 wk)

Examination schedule

|  | | observation | initial | 12 wk | 26 wk* | 52 wk* |
| --- | --- | --- | --- | --- | --- | --- |
| Acceptable range of gaps | |  |  | +4 wk | ± 4wk | ± 4wk |
| Informed consent | | **●** |  |  |  |  |
| Patient background | | **●** | |  |  |  |
| Serum lipids | TC |  | **●** | **●** | **●** | **●** |
|  | TG |  | **●** | **●** | **●** | **●** |
|  | LDL-C |  | **●** | **●** | **●** | **●** |
|  | HDL-C |  | **●** | **●** | **●** | **●** |
|  | RLP-C |  | **●** | **●** | **●** | **●** |
|  | Apo A-1 |  | **●** | **●** | **●** | **●** |
|  | Apo B |  | **●** | **●** | **●** | **●** |
|  | Apo E |  | **●** | **●** | **●** | **●** |
|  | MDA-LDL** |  | **●** | **●** | **●** | **●** |
|  | small dense LDL |  | **●** | **●** |  | **●** |
| Glucose metabolism | FPG |  | **●** | **●** | **●** | **●** |
|  | **IRI^＃^** |  | **●** | **●** | **●** | **●** |
|  | HbA1c |  | **●** | **●** | **●** | **●** |
| Inflammatory markers | hs-CRP |  | **●** | **●** |  | **●** |
| biochemistry | ALT、AST、γ-GTP、CPK、serum creatinine |  | **●** | **●** | **●** | **●** |
| Adverse events | |  | **●** | | | |

●, compulsory; *, for feasible patients only; **, for patients with CHD history only;

＃, measurement depends on the doctor’s decision in patients injecting insulin.

9. Criteria for terminating treatment

The drug treatment will be terminated and proper treatment will be dispensed to ensure safety when doctors accept the need in the following cases:

1. The subject retracts his or her enrollment or consent.
2. Lack of eligibility is proved after registration.
3. Change of residence or hospital, or failure to appear for a hospital visit.
4. Continuation of the study drugs is judged to be unfavorable because of an exacerbation of the original disease, a freak accident, or adverse events.
5. The doctors decide to discontinue the trial for other reasons.

10. Handling of adverse events

(1) Handling of the subjects in case of adverse events

The sub-investigators must properly treat adverse events and describe the events and treatment precisely in the medical records and case report forms. They must also inform the subjects of the need to treat adverse events and the cessation of study drugs when cessation is judged necessary.

(2) Report of severe adverse events

If severe adverse events occur, the sub-investigators must report them promptly to the director of the hospital.

Definition of severe adverse events

1. Adverse events leading to death
2. Life-threatening adverse events (events that put patients at risk of death)
3. Adverse events that require hospital admission or extension of hospitalization
4. Adverse events leading to permanent or remarkable disability/dysfunction
5. Events that cause congenital anomalies or birth anomalies
6. Other severe medical events *

* Medically critical events that require treatments for preventing these outcomes may be considered severe even if they are not currently life-threatening or do not currently merit admission. Such events would include, for example, allergic bronchitis requiring critical care in an emergency room or at home, or blood diseases or local paralysis not currently severe enough to merit admission.

The doctors must report every severe adverse event that occurs during the study period and every severe event that is later suspected to be associated with the study drugs.

11. Reporting on deviations from the protocol

(1) The sub-investigators should not deviate from the protocol or change the protocol without the prior consent of the representative investigator or an authorization from the hospital director based on prior screening at the Institutional Review Boards.

(2) The sub-investigators may deviate from the protocol or change the protocol without the prior consent of the representative investigator or prior screening at the Institutional Review Boards when there are compelling reasons, such as emergency avoidance. In such a case, the doctors must inform the representative investigator and Institutional Review Boards of the details of the change and explain why the change was necessary, in order to the obtain approval of the representative investigator, Institutional Review Boards, and hospital director retroactively.

(3) In case of a deviation from the protocol, the sub-investigators must record every detail of the deviation, explain the necessity, and deliver the record to the director or representative investigator in the prescribed format. The sub-investigators must also preserve a duplicate of this record.

12. Termination, cessation, or suspension of the trial

(1) Termination of the trial

The sub-investigators must turn in the trial termination report to the hospital director.

(2) Cessation or suspension of the trial

The sub-investigators will deliberate whether it will be possible to continue the trial in the following cases. If they judge it to be necessary to cease or suspend the trial, they will report the same promptly to the hospital director:

1. The emergence of crucial new information about the quality, safety, or efficacy of the study drugs.
2. Difficulty in recruiting subjects up to the predetermined number.
3. Difficulties with changes in the study design directed by the Institutional Review Boards.

13. Study period

From July 1, 2009 to June 30, 2013 (registration deadline June 30, 2012)

14. Data collection and statistical analysis

(1) data collection

The sub-investigators at each institution will fill in the survey slips and send them to the secretariat. The secretariat will tally the data by entering them in Excel files.

(2) definition of the analysis sets

Following are the definitions of the datasets to be used for the data analysis:

| Analysis sets | definition | analysis |
| --- | --- | --- |
| Full analysis sets  Efficacy by ITT（Intention To Treat） | All cases who have given consent and are registered, excluding cases not given the drugs | Background, efficacy, safety |
| Analysis sets fitting the protocol  Efficacy by PPS (Per Protocol Set) | Cases with the minimum compulsory program and available values for major variables, and without crucial contraventions of the protocol | Background, efficacy, safety |
| Analysis sets for safety | Prescribed cases with at a least single evaluation for safety | Background, safety |

(3) Statistical analysis

1) analysis for efficacy

The Wilcoxon rank sum test will be performed to analyze the percentage change in the level of serum LDL-C from baseline to week 12 of treatment.

The percentage of patients who reach the treatment goals at the end of the administration period will be calculated.

Wilcoxon signed rank test will be performed to analyze the change of examination data within each group.

2) The number and frequency of adverse effects will be calculated for subjects who receive at least a single administration of the study drugs. The same counting approach will be used for side effects.

15. Target number of cases and basis for setting it

(1) Target number of cases

Number of participating institutions: 25 institutions

Target number of cases: 120 cases

(2) Basis for setting the target number of cases

The first statin dose is assumed to account for 11.7% of the LDL-C-lowering effect in the double-dose statin administration group and 21.7% of the LDL-C-lowering effect in the ezetimibe add-on group. The SD is assumed to be 15 %, with an α of 0.05 and power of 90 %. Hence, there will be 60 cases per treatment group, assuming a dropout rate of 20 %.

16. Safety, human rights, and disadvantages faced by the study subjects

(1) Steps taken to protect human rights (Protection of privacy)

The confidentiality of the subjects must be duly considered when handling raw data for the study implementation, the consent form, etc. When case reports are to be submitted out-of-hospital, they will be handled using subject identification codes or the like. When the results of tests are published, the information will be screened to ensure that it cannot be used to identify the subjects. It will also be assumed, from the outset, that data on the subjects will not be used for any purposes other than the study.

17. Cost burdens on the patients

The study drugs administered are covered by national health insurance in Japan. Patients will bear the self-pay portion of the health insurance examination. The cost for the investigation of the original disease will also be covered by medical insurance. However, funds must be provided to cover research expenses for measuring LDL-C, RLP-C, small dense LDL, and hs-CRP, to ensure that the patients will bear no cost burdens by participating in the study.

18. Insurance coverage and compensation for health problems resulting from the study

(1) Compensation for health damage

If any health damage is caused as a result of participation in this study during treatment at this hospital, an application will be submitted for exemption of the patient’s share of the resulting medical expenses. Such an exemption will not apply, however, to the following costs.

1. Cost of medical care that is usually administered to treat complications and underlying disease
2. Costs for treatment of health damages caused by commercially available drugs given within their indications
3. Costs for treatment when liability claims are brought on the grounds of medical malpractice, etc.
4. Insurance coverage

The investigators and sub-investigators will take out liability insurance.

19. Observance of the Declaration of Helsinki and GCP

This study complies with the "Ethical Guidelines for Clinical Research" (459 Ministry of Health, Labour and Welfare Notification No. 2004) and the "Declaration of Helsinki" (including the 2004 annotated version).

20. Storage of records

The investigators are required to store documents used for the practice of this study and to discard them five years after the publication of the study (e.g., copies of application documents, notification documents from the hospital director, copies of the various application forms and reports, registration forms, copies of consent forms, case report forms, records or documents required to ensure the reliability of the other data).

21. Publication of research results

With regard to the publication of this study, the investigators and sub-investigators will decide a publication time, a presenter, presentation methods, and the like after the clinical research is completed.
